# D_2_O-Enabled Chemical Beaconing: Tracking Steroid Metabolism by Pooled Gut Microbiota

**DOI:** 10.3390/ijms27146466

**Published:** 2026-07-21

**Authors:** Boris Tupertsev, Anna Vishnevskaya, Tatiana Ikonnikova, Yury Kostyukevich

**Affiliations:** 1 Center for Bio- and Medical Technologies, Nobel Str., 3, 121205 Moscow, Russia; btoupersev@gmail.com (B.T.); ai.vish@yandex.ru (A.V.); ikonnikova_tanya@mail.ru (T.I.); 2 Moscow Center for Advanced Studies, Kulakova Str. 20, 123592 Moscow, Russia

**Keywords:** deuterium oxide, stable isotope labeling, steroid metabolism, gut microbiota, HPLC-HRMS, microbial biotransformation

## Abstract

The gut microbiota actively metabolizes steroid hormones, but the mechanisms of these transformations—particularly the sequence of enzymatic reactions and the source of hydrogen atoms—remain poorly understood. Conventional analytical approaches are hampered by the complex fecal matrix, isomeric metabolites, and the lack of authentic reference standards. Here we present a strategy based on parallel incubation of steroids with pooled human gut microbiota in H_2_O and D_2_O, followed by HPLC-HRMS, with the aim of determining the sequence of reductive steps and tracking the incorporation of atoms into steroid metabolites. Using a pooled fecal inoculum from multiple donors (*n* = 18) and three steroid substrates, we demonstrate that the characteristic mass shift (1.0063 Da per deuterium atom) enables detection of metabolites and distinguishes multi-step enzymatic reactions in the microbial community without recombinant enzymes and authentic standards. Progesterone and 19-hydroxy-4-androstene-3,17-dione underwent sequential two-step reduction incorporating up to three deuterium atoms, while 17α-hydroxypregnenolone followed a three-step pathway incorporating up to four deuterium atoms, consistent with localization to the A-ring. This workflow provides a practical tool for investigating gut microbial steroid metabolism in the context of human physiology and disease, with potential applications in monitoring microbial activity in endocrine disorders, inflammatory bowel disease, and neuropsychiatric conditions.

## 1. Introduction

The intestinal microbiota is a complex ecosystem with more than 1000 bacterial species, functioning as a distinct “metabolic organ” capable of modifying a wide range of xenobiotics, including drugs [1,2].

The scale of this phenomenon was systematically characterized by Zimmermann et al., who tested 76 gut bacterial species against 271 orally administered drugs [3]. Two-thirds of the drugs were metabolized by at least one strain, with individual strains transforming up to 176 compounds. Importantly, they identified bacterial genes responsible for metabolizing steroid compounds, including progesterone. This steroid hormone and its synthetic analogs (levonorgestrel, medroxyprogesterone acetate) are widely used in reproductive medicine but suffer from low oral bioavailability (~10%) [4], traditionally attributed to hepatic and intestinal CYP450 metabolism [5,6].

Delivering progestogens to the colon—where CYP activity is low—is an attractive strategy, but the colonic microbiota rapidly metabolizes these hormones. In a recent study, the authors examined the stability of three progestogens in a human fecal inoculum and showed that progesterone disappears within 2 h (half-life 28 min) but could not identify the transformation products [7].

A recent preprint using a multi-omics approach—HPLC-HRMS-based untargeted metabolomics combined with metagenomic sequencing—analyzed fecal samples from women receiving progesterone and confirmed that gut microbiota converts progesterone to neuroactive steroids (primarily pregnanolone) and proposed enzyme families and associated bacterial taxa [8]. Nevertheless, the study did not capture intermediate metabolites, validate the proposed enzymes using individual isoforms, characterize formation kinetics, or determine the source of reducing hydrogen atoms.

Parallel progress has been made by Arp et al. in systematically characterizing three key enzyme families responsible for steroid hormone biotransformation in the gut—Δ^4^-3-ketosteroid 5β-reductase, 3β-hydroxysteroid dehydrogenase/Δ^5−4^ isomerase, and Δ^6^-3-ketosteroid reductase—using recombinant enzymes and metagenomics combined with HPLC-HRMS-based metabolite annotation of strain-level incubations with reference standards [9]. Additionally, the OsrABC reductive pathway in *Clostridium steroidoreducens* has been characterized, and homologs of these genes are enriched in Crohn’s disease patients and reduce prednisolone bioavailability in mice [10]. This underscores the clinical relevance of microbial steroid metabolism and positions steroids within the broader class of bioactive lipids whose biotransformation by the gut microbiota can influence host physiology.

Disruption of microbial steroid metabolism has been increasingly linked to human pathologies. In polycystic ovary syndrome, alterations in gut microbiota composition and serum steroid profiles are associated with hyperandrogenism, suggesting a role for microbial steroid conversion in disease phenotypes [11]. In Cushing’s syndrome, gut dysbiosis with enrichment of cortisol-degrading bacteria may contribute to the regulation of host cortisol levels, potentially influencing disease progression and treatment response [12]. In inflammatory bowel disease, gut bacteria can inactivate glucocorticoids via the reductive OsrABC pathway, which may compromise the efficacy of steroid-based therapies [10]. Furthermore, gut bacterial production of neuroactive steroids has been implicated in postpartum depression and other neuropsychiatric disorders, highlighting a gut–brain axis mediated by microbial steroid metabolism [13]. These findings underscore the clinical need for robust analytical tools to dissect the enzymatic steps and substrate specificities of microbial steroid transformations.

Nevertheless, such approaches are resource-intensive, requiring strain isolation, metagenomic sequencing, and authentic standards for each potential metabolite. They are difficult to scale for multiple substrates or donor samples and do not readily capture the network effects of a complex microbial community where multiple enzymes and strains act simultaneously.

Unlike resource-intensive strain- or enzyme-level studies, *ex vivo* incubations with gut microbiota offer a practical alternative: they are higher in throughput, lower in cost, and capture community-level metabolism, where multiple bacterial species and enzymes act simultaneously on a given substrate [7,8,14]. Pooling samples from multiple donors further reduces interindividual variability, making this approach a common practice in drug metabolism studies [15,16,17,18,19,20].

However, even with pooled inocula, the system remains complex. Sequential reactions involving multiple enzyme isoforms produce isomeric products (5α/5β, 3α/3β) that are indistinguishable by conventional MS/MS [16]. Chiral chromatography is rapidly fouled by fecal matrix, and no universal chiral column exists for all analyte–matrix combinations [21]. Moreover, reference standards for most potential steroid metabolites are commercially unavailable.

Stable isotope labeling, particularly deuterium (^2^H, D) and oxygen-18 (^18^O), offers a powerful strategy to overcome these analytical challenges. Although it cannot resolve optical isomerism, it effectively addresses other limitations such as the lack of reference standards and the difficulty of detecting metabolites in complex fecal matrices. Several chemical and enzymatic approaches for stable isotope labeling exist, including NAD(P)H-dependent [22] and non-NAD(P)H-dependent methods [23]. Gaseous ^18^O_2_ and H_2_^18^O have been used with enzymes to study mechanisms, identify the source of atoms, track metabolite formation, and distinguish them from other compounds in complex samples [24,25,26]. Some approaches rely on reversible isotope exchange reactions, such as H/D exchange at heteroatoms or ^16^O/^18^O exchange for carbonyl and carboxyl groups; however, other reactions incorporate labeling at stable positions (e.g., C–D bonds) [23]. In the context of the anaerobic gut environment, reductive enzymes dominate, making deuterium oxide (D_2_O) an ideal labeling agent. When steroid substrates are incubated in D_2_O, deuterium can be incorporated into reaction products either through isotope-labeled cofactors [22] or directly from the solvent via enzyme-catalyzed exchange [23]. Parallel incubations in H_2_O and D_2_O produce a characteristic mass shift for deuterium-containing metabolites: each incorporated deuterium atom adds approximately 1.0063 Da to the molecular mass. This mass difference allows unambiguous detection of metabolites against the fecal background without the need for authentic reference standards. Moreover, the pattern of deuterium incorporation provides mechanistic information: reduction of double bonds introduces stable C–D bonds that remain during sample workup, whereas reduction of ketones introduces exchangeable O–D bonds that are lost, and isomerization does not incorporate deuterium. Tracking the number and retention of deuterium atoms thus reveals the sequence of enzymatic reactions, localizes reduction sites to specific regions of the steroid nucleus, and identifies the source of hydrogen atoms—information that is inaccessible to conventional metabolomics approaches.

Thus, the aim of this study was to apply this D_2_O-based labeling strategy to three representative steroid substrates,: progesterone, 19-hydroxy-4-androstene-3,17-dione, and 17α-hydroxypregnenolone, in order to analyze the sequence of reductive transformations catalyzed by the pooled human gut microbiota and identify the source of the hydrogen atoms introduced at each stage of fermentation.

## 2. Results

### 2.1. Optimization of Labeling Conditions and General Observations

**Figure 1:**
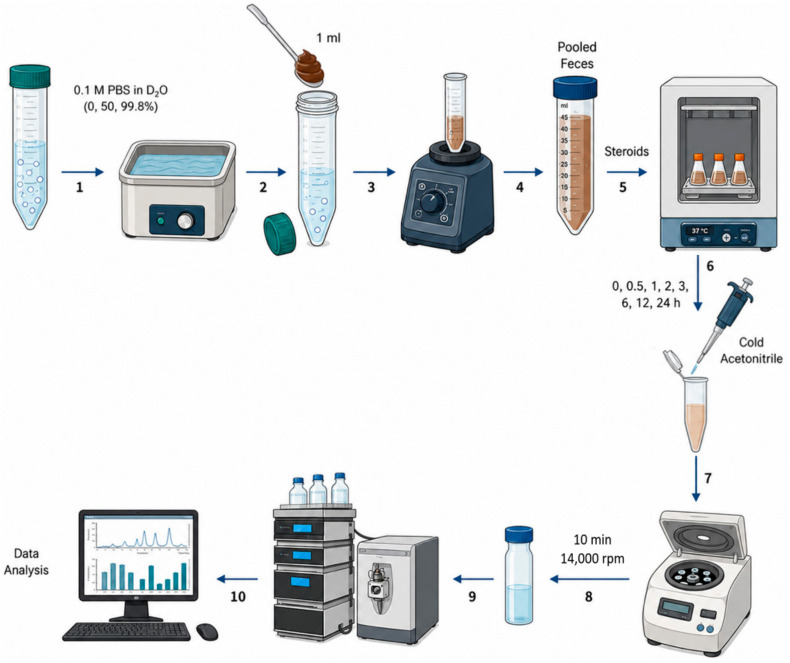
Experiment design. 1—PBS buffer degassing; 2—addition of fecal samples to PBS cushion; 3—intensive shaking of PBS with feces mixture; 4—pooled feces from 18 donors with PBS at a 1:1.5 (*v*/*v*) fecal-to-buffer ratio; 5—addition of steroid stock solution; 6—anaerobic incubation at 37 °C; 7—addition of cold acetonitrile to stop the reaction; 8—centrifugation and transfer to HPLC vials; 9—HPLC-HRMS analysis; 10—data analysis.

The general scheme of the experiment is shown in Figure 1.

**Figure 2:**
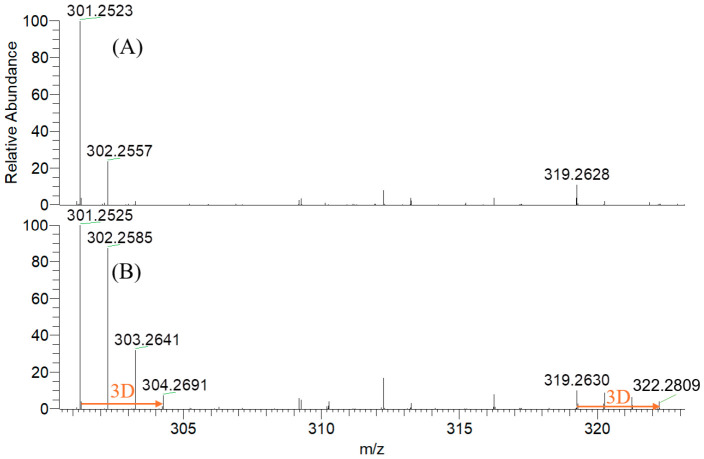
MS of a compound with a retention time of 1 min in the mode of positively charged ion registration: (**A**) during incubation in H_2_O; (**B**) during incubation in D_2_O.

Initial experiments with progesterone in 50% D_2_O showed complete substrate depletion within 3 h, comparable to the H_2_O, indicating no significant kinetic isotope effect at this D_2_O concentration. Although MS/MS spectra were not obtained in untargeted DDA mode due to weak ionization, in-source fragmentation, and matrix effects, the labeling approach enabled detection of potential metabolites based on the characteristic mass shift of 1.0063 Da per deuterium atom by Equation (1). In H_2_O, ions at *m*/*z* 301.2523 Da (C_21_H_33_O, −1.0 ppm) were among the most intense signals; in D_2_O, these corresponded to ions with 0–3 deuterium atoms at *m*/*z* 301.2525–304.2714 Da (C_21_H_33_O-C_21_H_30_D_3_O, −0.3 ppm). The corresponding parent ions were observed at *m*/*z* 319.2628 (C_21_H_35_O_2_, −1.1 ppm) in H_2_O and at *m*/*z* 319.2630–322.2809 Da (C_21_H_35_O_2_-C_21_H_32_D_3_O_2_, −3.4 ppm) in D_2_O (Figure 2). The observed signals presumably correspond to pregnanolones, consistent with the literature [9].

However, in isotopic clusters from the 50% D_2_O incubation, the intensity of the unlabeled signal remained among the highest, indicating a low degree (LD 55%) of labeling despite the incorporation of up to three deuterium atoms (N_D_). To maximize labeling efficiency, subsequent incubations were performed in 99.8% D_2_O. Under these conditions, the metabolic rate decreased (complete metabolism by 6 h instead of 3 h), while the degree of labeling increased by LD 90%, enabling unambiguous detection of metabolites. The pH, incubation temperatures, and PBS concentration were kept constant, as these conditions are critical for maintaining bacteria activity. To study the applicability if the approach, two additional steroids—19-hydroxy-4-androstene-3,17-dione and 17α-hydroxypregnenolone—were tested alongside progesterone. Their metabolism was analyzed under the optimized conditions: anaerobic atmosphere, 37 °C, aqueous 0.1 M PBS (0, 99.8% of D_2_O), pH 6.8, with time points 0, 0.5, 1, 2, 3, 6, and 12 h.

### 2.2. Metabolism of Progesterone by Pooled Gut Microbiota

**Figure 3:**
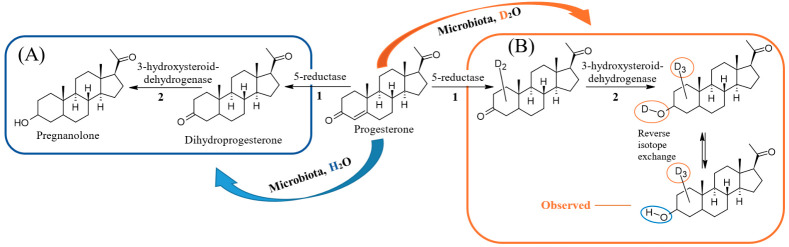
Metabolic map of progesterone during incubation with gut microbiota: (**A**) in aqueous PBS; (**B**) in PBS containing 99.8% D_2_O. Proposed sites of deuteration are shown in orange; sites of reverse isotope exchange are shown in blue.

Comparison of H_2_O and D_2_O incubations of progesterone revealed three metabolic features in D_2_O exhibiting characteristic isotopic shifts of 1.0063 Da per deuterium atom (Figure 3, Table 1).

#### 2.2.1. Identification of Pregnanolone Isomers

**Figure 4:**
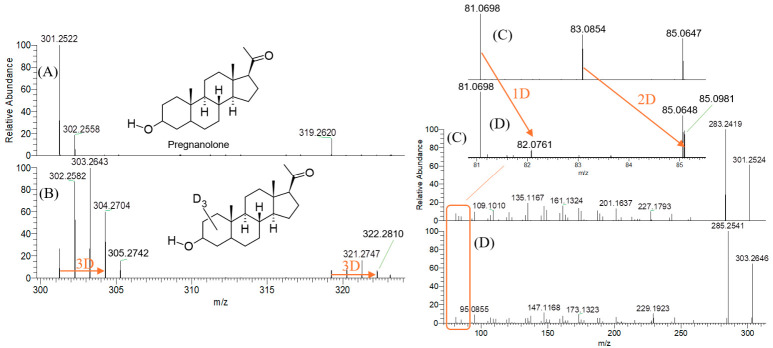
The mass spectra of the compound with R_t_ 9.55 (similar for the compound with R_t_ 9.79 min): (**A**) MS during incubation in H_2_O; (**B**) MS during incubation in 99.8% D_2_O; (**C**) MS/MS of ion with *m*/*z* 301.2524 Da during incubation in H_2_O; (**D**) MS/MS of ion with *m*/*z* 303.2646 Da during incubation in 99.8% D_2_O.

Metabolites at R_t_ 9.55 and 9.79 min with *m*/*z* 319.2620 Da (C_21_H_35_O_2_, −3.6 ppm) in H_2_O corresponded to the same retention times in D_2_O at *m*/*z* 319.2632–322.2809 Da (C_21_H_35_O_2_—C_21_H_32_D_3_O_2_, −3.4 ppm). The two distinct retention times (9.55 and 9.79 min) for the same nominal mass indicate the formation of isomeric products, consistent with published data on pregnanolone stereoisomers generated by gut bacterial reductases and/or hydroxysteroid dehydrogenases [9]. The specific stereochemical configuration of each peak could not be assigned without chiral chromatography. PRM MS/MS spectra from fivefold concentrated samples confirmed these signals as pregnanolones [9] and localized deuterium labels to the A-ring: *m*/*z* 81.0698 (C_6_H_9_, −1.0 ppm) shifted to 82.0761 (C_6_H_8_D, −0.6 ppm); *m*/*z* 83.0854 (C_6_H_11_, −1.5 ppm) shifted to 85.0981 (C_6_H_9_D_2_, 0.2 ppm). No shift was observed for *m*/*z* 85.0648 (C_5_H_9_O, 0.1 ppm), corresponding to D-ring and acetyl group fragments (Figure 4).

#### 2.2.2. Identification of an Intermediate Metabolite Dihydroprogesterone

**Figure 5:**
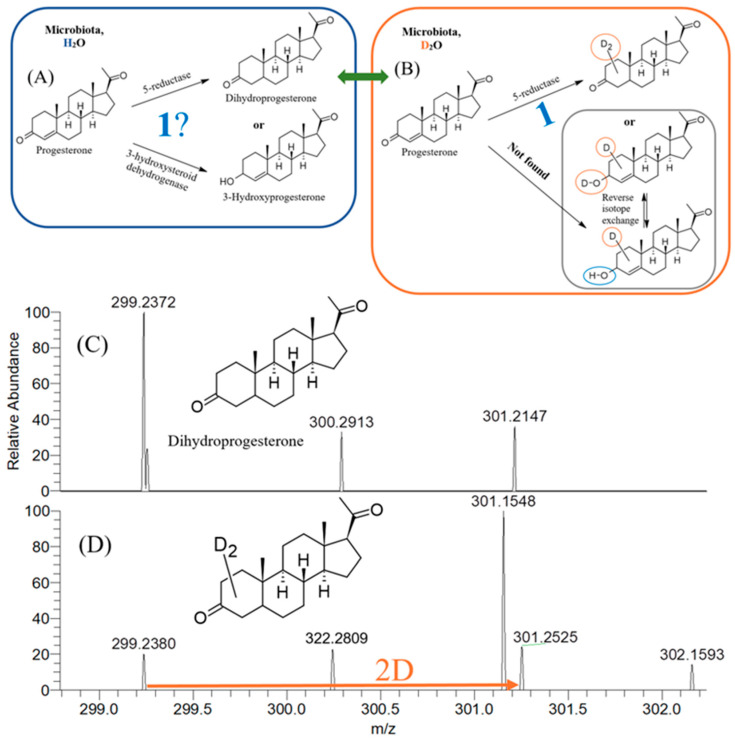
Determination of the intermediate of progesterone metabolism by the intestinal microbiota: (**A**) possible structures of metabolites corresponding to the signal with *m*/*z* 299.2370 Da during incubation in H_2_O; (**B**) the detected product during incubation in D_2_O; (**C**) the MS of the intermediate product detected during incubation in H_2_O; (**D**) the MS of the corresponding intermediate product during incubation in 99.8% D_2_O.

In H_2_O, a single signal was detected at R_t_ 9.88 min with *m*/*z* 299.2372 Da (C_21_H_31_O, 0.9 ppm), a fragment ion derived from [M+H]^+^ at *m*/*z* 317.2475 (C_21_H_33_O_2_). This signal could correspond to either dihydroprogesterone or 3-hydroxyprogesterone (Figure 5). In D_2_O, this signal expanded to ions at *m*/*z* 299.2380–301.2525 Da (C_21_H_31_O-C_21_H_29_D_2_O, 9.8 ppm) (Figure 5). The presence of two deuterium labels supports the identification of this intermediate as dihydroprogesterone (reduction of the C4–C5 double bond). In contrast, 3-hydroxyprogesterone would contain one exchangeable O–D bond, which would be lost during sample workup [27].

### 2.3. Metabolism of 19-Hydroxy-4-Androstene-3,17-Dione by Pooled Gut Microbiota

**Figure 6:**
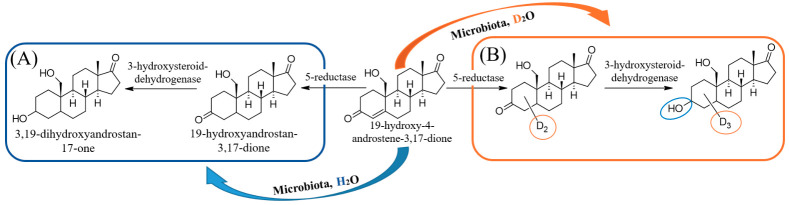
Metabolic map of 19-hydroxy-4-androstene-3,17-dione during incubation with gut microbiota: (**A**) in aqueous PBS; (**B**) in PBS containing 99.8% D_2_O. Proposed sites of deuteration are shown in orange; sites of reverse isotope exchange are shown in blue.

The method enable detection of three metabolites of 19-hydroxy-4-androstene-3,17-dione via isotopic shifts (Figure 6, Table 2).

An intermediate metabolite, tentatively identified as 19-hydroxyandrostan-3,17-dione, contained up to two deuterium labels, as evidenced by characteristic shifts from *m*/*z* 305.2112 (C_19_H_29_O_3_, 0.3 ppm) in H_2_O to *m*/*z* 307.2230 (C_19_H_27_D_2_O_3_, −2.2 ppm) in D_2_O at R_t_ 5.07 min (Figure S1).

The major metabolites, tentatively identified as 3,19-dihydroxyandrostan-17-ones, contained up to three deuterium labels in D2O at *m*/*z* 289.2160–292.2339 Da (C_19_H_29_O_2_-C_19_H_26_D_2_O_2_, −3.9 ppm). As observed for progesterone, deuterium was localized to the A-ring, as confirmed by characteristic shifts in MS/MS spectra obtained from fivefold concentrated samples: *m*/*z* 93.0697 (C_7_H_9_, −1.9 ppm) to 94.0759–95.0824 (C_7_H_8_D-C_7_H_7_D_2_, −2.7 ppm), among others (Figure S2).

### 2.4. Metabolism of 17α-Hydroxypregnenolone by Pooled Gut Microbiota

**Figure 7:**
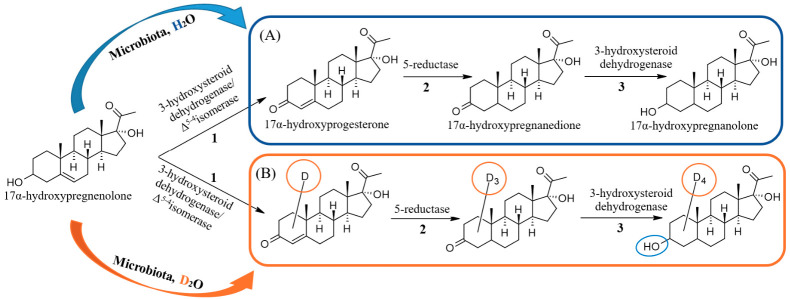
Metabolic map of 17α-hydroxypregnenolone during incubation with gut microbiota: (**A**) in aqueous PBS; (**B**) in PBS containing 99.8% D_2_O. Proposed sites of deuteration are shown in orange; sites of reverse isotope exchange are shown in blue.

The method enabled detection and identification of four metabolites (Figure 7, Table 3).

**Figure 8:**
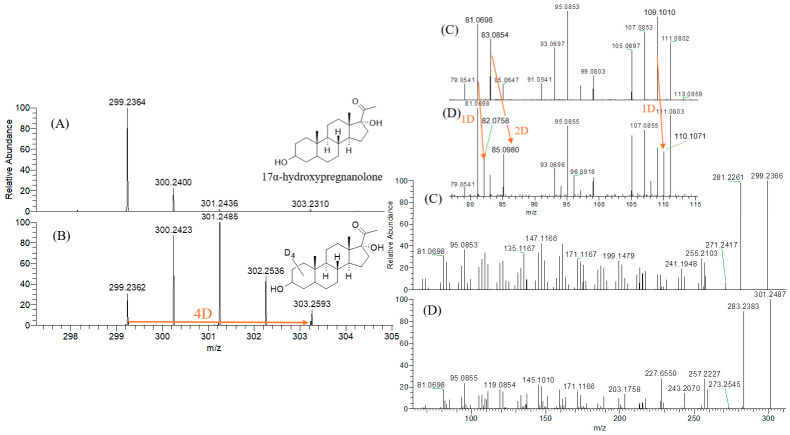
The mass spectra of the compound with RT 8.64 (similar for the compound with R_t_ 8.13 min). (**A**) MS during incubation in H_2_O; (**B**) MS during incubation in D_2_O; (**C**) MS/MS of ion with *m*/*z* 299.2366 Da during incubation in H_2_O; (**D**) MS/MS of ion with *m*/*z* 301.2485 Da during incubation in 99.8% D_2_O.

Unlike the incubations of progesterone and 19-hydroxy-4-androstene-3,17-dione, where the final products contained up to three deuterium atoms (N_D_ according to Equation (1)), the products of 17α-hydroxypregnenolone—tentatively identified as 17α-hydroxypregnanolones—contained up to four deuterium labels at *m*/*z* 299.2364–303.2593 Da (C_21_H_31_O-C_21_H_27_D_4_O, −9.1 ppm) (Figure 8). Characteristic shifts of fragment ions localized the labels to the A-ring: *m*/*z* 81.0698 (C_6_H_9_, −1.0 ppm) to 82.0758 (C_6_H_8_D, −4.3 ppm); *m*/*z* 83.0854 (C_6_H_11_, −1.5 ppm) to 85.0981 (C_6_H_9_D_2_, 0.2 ppm), among others. This indicates more complex biotransformations than direct reduction of the B-ring of the parent steroid.

Further analysis revealed putative intermediates. 17α-hydroxyprogesterone was detected at R_t_ 8.41 min with *m*/*z* 313.2167 Da (C_21_H_29_O_2_, 1.6 ppm), corresponding to signals at *m*/*z* 313.2153–314.2205 Da (C_21_H_29_O_2_-C_21_H_28_DO_2_, −6.3 ppm) in D_2_O (Figure S3). Additionally, 17α-hydroxypregnanedione was detected at *m*/*z* 297.2214 (C_21_H_29_O), similar to the ionized form of the parent compound but with a retention time of 8.21 min (Figure S4). Notably, the parent steroid showed no labeling in D_2_O, whereas this putative metabolite corresponded to signals at *m*/*z* 297.2200–300.2410 Da (C_21_H_29_O-C_21_H_26_D_3_O, −4.3 ppm).

### 2.5. General Observation on Reaction Kinetics in 99.8% D_2_O

For all three steroids tested, similar kinetic patterns were observed. The intermediate metabolites appeared within 30 min in both H_2_O and 99.8% D_2_O. In H_2_O, they were no longer detectable after 1 h, whereas in 99.8% D_2_O they persisted for up to 3 h. The final metabolites were already detectable at 30 min, and their concentrations increased continuously up to 3 h in H_2_O and up to 6 h in D_2_O. Thus, the reduction reactions were consistently slower in deuterated water, indicating a modest kinetic isotope effect under the conditions tested.

## 3. Discussion

A key finding of this study is that metabolites could be detected even when MS/MS spectra were unavailable during untargeted DDA mode due to low ionization efficiency and matrix effects. The characteristic mass shift (Equation (1)) between H_2_O and D_2_O incubations enabled their unambiguous identification against the complex fecal background. Furthermore, the number and positions of incorporated deuterium atoms revealed the sequence of enzymatic reactions in this biologically rich environment—where multiple bacterial species and enzyme families act simultaneously—and allowed the detection of minor metabolites. This information would be difficult or impossible to obtain using conventional incubations and HPLC-HRMS approaches.

Our data not only confirm the previously described metabolism and end-products identified by Coombes et al. [7] and Brandon-Mong et al. [8] but also provide the first direct tracing of hydrogen atom sources, revealing the specificity and sequence of each reductive step. Furthermore, the D_2_O-labeling workflow described here is not limited to the metabolism of the three tested steroids. It can be applied to other steroid hormones and bioactive lipids, offering a route to investigate how microbial biotransformation alters their biological activity. This may be relevant for understanding the role of gut microbiota in steroid-related disorders, although direct clinical correlations remain to be established.

Nevertheless, several limitations of the D_2_O-labeling approach should be acknowledged. First, while the method reliably distinguishes between double-bond and ketone reduction and localizes deuterium incorporation to the A-ring based on characteristic MS/MS fragments, it does not resolve stereochemical configurations (5α/5β or 3α/3β epimers) without chiral chromatography. Our assignment of enzymatic activities (e.g., 5β-reductase vs. 5α-reductase) therefore relies on the known regioselectivity of gut microbial steroid reductases reported by Arp et al. [9] rather than on direct chiral analysis. Second, metabolite identification was performed by MS/MS spectral matching against databases and literature data (MS and MS/MS), as authentic standards for most potential steroid metabolites were not commercially available. While D_2_O labeling provided orthogonal confirmation of reduction sites, full structural confirmation would require synthetic reference compounds. Third, the degree of deuterium incorporation reached approximately 90% in 99.8% D_2_O, but not 100%; thus, the observed number of deuterium atoms may underestimate the true labeling due to in-source fragmentation, isotopic overlap, or low signal intensity for minor isotopologues. Fourth, while pooled fecal inoculum effectively reduces interindividual variability, it may dilute strain-specific metabolic activities that could be relevant for certain donors, potentially masking donor-dependent metabolic idiosyncrasies. The modest kinetic isotope effect observed in 99.8% D_2_O (complete metabolism in 6 h vs. 3 h in H_2_O) indicates that reaction rates are altered under labeling conditions, although the pathway and product distribution remained consistent. Finally, selective detection via the characteristic mass shift facilitates identification against the fecal background, yet low-abundance intermediates may still escape detection due to matrix suppression, and the proposed enzyme activities remain correlative—direct validation by proteomics or enzyme activity assays would strengthen the mechanistic conclusions. Despite these limitations, the D_2_O-labeling workflow provides unique mechanistic insights that are inaccessible by conventional metabolomics and offers a scalable platform for studying microbial biotransformations.

### 3.1. Mechanistic Dissection of Progesterone and 19-Hydroxy-4-Androstene-3,17-Dione Reduction by the Pooled Microbiota

Our data are consistent with a sequential two-step reduction, where reduction of the C4–C5 double bond precedes reduction of the C3 carbonyl. This conclusion is supported by the detection of a dihydro-intermediate containing exactly two stable deuterium atoms (C–D bonds), which distinguishes it from a hypothetical 3-hydroxy intermediate that would contain only one exchangeable deuterium.

First, the rapid and complete conversion of progesterone to a dihydro-intermediate containing exactly two stable deuterium atoms (Table 1) demonstrates reduction of the C4–C5 double bond. If reduction had occurred at the C3 carbonyl first, the intermediate would have contained only one stable C–D bond plus an exchangeable O–D, which would not survive the analytical workflow. The presence of exclusively two labels thus serves as a “chemical fingerprint” for the activity of 5β-reductase.

Recent breakthroughs by Arp et al. have identified the specific gut bacterial enzymes responsible for this step—Δ^4^-3-ketosteroid 5β-reductase [9]. Such a reduction, which incorporates two hydrogen atoms (or deuterium atoms, if the reaction occurs in a D_2_O-enriched environment) across the C4 and C5 positions, aligns with the known ene-reductase mechanism of the Old Yellow Enzyme family, to which these 5β-reductases belong [9].

Second, the subsequent conversion to the final product containing three deuterium atoms confirms the reduction of the C3-ketone to a hydroxyl group. Unlike the previous step, the third label is not always retained. The partial back-exchange of this deuterium (evidenced by the persistence of the unlabeled D_0_ species alongside the D_3_ species) is characteristic of a labile O–D bond. This proves that the C3 hydroxyl hydrogen is derived from the solvent (D_2_O) or is rapidly exchangeable post-catalysis, rather than being stereospecifically transferred from a cofactor as a fixed hydride, consistent with known mechanisms of ketosteroid reductases [28,29].

### 3.2. Reconstitution of the Δ^5^-3β-OH Pathway

The metabolism of 17α-hydroxypregnenolone revealed an even more complex transformation, confirming that the pooled microbiota possesses the full enzymatic arsenal for the complete activation and subsequent inactivation of steroid precursors [29].

The first step involves oxidation of the 3β-hydroxyl group to a 3-ketone, followed by isomerization of the double bond from the C5–C6 to the C4–C5 position. The immediate product, 17α-hydroxyprogesterone, incorporated a single deuterium atom, which is consistent with the activity of 3β-hydroxysteroid dehydrogenase/Δ^5−4^ isomerase (3β-HSD) [9]. Biochemical studies have shown that for 3β-HSD, the cofactor (NAD^+^) binds first, inducing a conformational change that activates the isomerase activity [30]. In a D_2_O environment, base-catalyzed enolization at the active site can lead to the exchange of a single proton at C2 or C4, explaining the single label in the product [29].

Once the Δ^4^-3-ketone is formed, the molecule enters the same reductive pathway as progesterone. Reduction of the C4–C5 double bond by 5β-reductase (the second step) adds two stable labels, resulting in the 17α-hydroxypregnanedione intermediate (D_0_–D_3_). Notably, this intermediate has the same *m*/*z* as the parent compound with no labeling during incubations. Reduction of the C3-ketone (the third step) adds a fourth label, yielding the 17α-hydroxypregnanolones (D_0_–D_4_) (Table 3).

## 4. Materials and Methods

### 4.1. Chemicals and Reagents

Progesterone, 19-hydroxy-4-androstene-3,17-dione, 17α-hydroxypregnenolone, phosphate-buffered saline (PBS) tablets, and LC-MSgrade solvents (acetonitrile, formic acid) were obtained from Merck KGaA (Darmstadt, Germany). Deuterium oxide (D_2_O, 99.8%, atom % D) was purchased from ST Selivanenko O.I. (Moscow, Russia). Water used for the preparation of aqueous buffers and LC-MS analysis was purified using a Milli-Q system (Millipore, Burlington, MA, USA).

### 4.2. Preparation of Pooled Fecal Microbiota Inoculum and Stocks

Pooled freshly obtained human fecal samples were used as the microbiota source. To reduce interindividual variability and create a standardized inoculum, samples from 18 healthy donors (9 males and 9 females, 18–50 years old) who had not taken antibiotics, bacterial drugs or fermented products (including dairy, yeast-fermented beverages, etc.) for two weeks prior to the experiment were combined.

The protocol was reviewed by the internal ethics committee of the Center for Bio- and Medical Technologies, which determined that formal approval was not required for this non-interventional study using anonymized biological material from healthy volunteers, in accordance with local guidelines. All donors provided verbal informed consent prior to sample collection. No identifiable personal data were recorded.

For each experiment (0%, 50% and 99.8% D_2_O enrichment) 0.1 M PBS (pH 6.8) was degassed for 15 min and thermostatted at 37 °C prior to use. A 50 mL Falcon tube was initially charged with a 3 mL cushion of PBS. Individual fecal samples were then rapidly aliquoted by volume using a 1 mL measuring spatula and directly combined into the tube to form a composite pool (18 mL of biomass total, added into the 3 mL PBS cushion). To ensure effective homogenization of the dense biomass and to standardize the final isotopic enrichment by accounting for the residual water content of the raw fecal matrix, the remaining volume of the same degassed and thermostatted PBS was added to achieve a final 1:1.5 (*v*/*v*) fecal-to-buffer ratio.

Stock solutions of the three test compounds (progesterone, 19-hydroxy-4-androstene-3,17-dione, 17α-hydroxypregnenolone) were prepared in acetonitrile at a concentration of 1 mg/mL.

### 4.3. Incubation Conditions

Parallel incubations were performed under anaerobic conditions at 37 °C in 0.1 M PBS (0, 50, 99.8% D_2_O), pH 6.8. Each test compound was incubated separately. For this, 1 mL of the pooled fecal inoculum was placed in a 2 mL tube, 20 μL of the corresponding steroid stock solution (1 mg/mL) was added, yielding a final concentration of 20 μg/mL (60–66 μM, depending on the substrate) in the incubation mixture. All incubations were performed in duplicate (*n* = 2) using independent pooled fecal preparations.

For kinetic studies, aliquots were withdrawn at specific time points. Based on initial results with progesterone in 50% D_2_O (complete metabolism within 3 h), the time points were set at 0, 0.5, 1, 2, 3, 6, and 12 h. Controls consisted of steroid incubated in sterile (without microbiota) PBS (0, 50, 99.8% D_2_O), and pooled feces without external steroids in PBS (0, 50, 99.8% D_2_O).

### 4.4. Sample Preparation

After incubation, the reaction was stopped by adding ice-cold acetonitrile (1:2 *v*/*v*), followed by vortexing (3 min) and centrifugation at 14,000 rpm to precipitate contaminants. For MS/MS analysis (parallel reaction monitoring, PRM), selected samples were concentrated fivefold under an atmosphere of N_2_.

### 4.5. High-Performance Liquid Chromatography/High-Resolution Mass Spectrometry (HPLC-HRMS) Conditions

All experiments were carried out with Dionex UltiMate 3000 coupled with QExactive Orbitrap (Thermo Fisher Scientific, Waltham, MA, USA). We used a Hypersil Gold aQ (2.1 × 150 mm, 1.8 µm) HPLC column with a guard column (Thermo Scientific). Mobile phase A involved 0.1% formic acid in 5% aqueous solution of acetonitrile. Mobile phase B involved 0.1% formic acid in acetonitrile delivered as the following gradient at a flow rate of 0.60 mL/min: 0–1.0 min 0% B, 1.0–16.0 min 5–95.0% B, 16.0–18.0 95% B, 18.0–18.3 min 95–0% B and 18.3–21.0 min 0% B. The injection volume was 3 µL. The column compartment temperature was set to 40 °C.

The HRMS resolving power was 35,000 (for *m*/*z* = 200). The Sheath, Aux and Spare gases were set to 45, 15 and 5, respectively. The Spray Voltage was 4.1 kV (for positive and negative ionization modes), the temperature of the desolvating capillary was 320 °C. S-Lens RF level was 50 and the source temperature was set to 200 °C. Open screening for metabolites was performed by Full MS followed (100–900 *m*/*z*) by data-dependent analysis (DDA) both in positive and negative ionization modes, and targeted metabolite searches were carried out by parallel reaction monitoring (PRM). Control samples included steroids in PBS (0, 50, 99.8% of D_2_O) and pooled fecal samples in PBS (0, 50, 99.8% of D_2_O), which were analyzed under the same conditions. Each incubation and control sample was injected in duplicate to ensure reproducibility. To prevent HPLC column contamination between samples, blank solutions of acetonitrile were analyzed.

### 4.6. Data Analysis

HPLC-HRMS spectral data were processed using Xcalibur™ 4.7 software. For targeted and untargeted metabolite identification and profiling, Compound Discoverer™ (version 3.3) was used (Thermo Fisher Scientific, Waltham, MA, USA).

The results from replicate incubations and duplicate injections were averaged for labeling degrees (LD) and deuterium counts. Candidate metabolites were manually verified by checking exact mass, interpreting MS/MS spectra according to fragmentation rules, and assessing the *m*/*z* shift according to Equation (1).

(1) ∆MH=ND×MD2−MH1=ND×(2.0141−1.0078)=ND×1.0063 Da

where ND is the number of incorporated deuterium atoms.

For additional structural confirmation, the obtained mass spectra were matched against NIST 23, Human Metabolome Database (HMDB, version 5.0), mzCloud (Thermo Fisher Scientific) and literature data.

To account for instrument error, limited resolution, and contributions from overlapping isotropic signals, deuterium labeling was considered present when the mass difference between two isotopic peaks fell within the range of *m*/*z* 1.0040–1.0080 Da. Ion annotation includes predicted molecular formulas and measured *m*/*z* errors in ppm indicated in brackets.

Additionally, due to minor chromatographic shifts between compounds and their analogues with varying degrees of LD, mass spectra were averaged across the elution range of each analyte to ensure consistent isotopic profiling.

To quantify isotope incorporation we introduced the Labeling Degree (LD) metric:

(2) $$LD=\frac{\sum {Area}_{LI}}{{Area}_{NLI}+\sum {Area}_{LI}}\times 100\%$$

where LI—labeled ion, NLI—non-labeled ion. Areas for LD calculation were selected in Base Peak mode of Qual Browser (Xcalibur™) based on the theoretical monoisotopic mass with a precision of four decimal places. Signal search was performed with a mass tolerance of 5 ppm, and the area was considered valid only if the observed mass shift between isotopic peaks met the criteria for deuterium labeling.

Since mass spectrometry does not allow the precise determination of deuterium positions, only the steroid ring to which the label is attached (e.g., the A-ring) is indicated in this work. Likewise, because no chiral columns were used, the description of compounds and presumed enzymes is given without specifying the optical configuration. The number of isotopic labels and LD values determined by mass spectrometry may differ from the actual number in the structure due to in-source fragmentation with loss of labels or low signal intensity, which may prevent detection of all incorporated deuterium atoms.

## 5. Conclusions

This study demonstrates that D_2_O-based stable isotope labeling coupled with HPLC-HRMS is a robust, compound standard and recombinant enzyme-free strategy for dissecting steroid metabolism by complex gut microbial communities. By monitoring the characteristic mass shift between H_2_O and D_2_O incubations, metabolites were detected in highly loaded matrices even when MS/MS spectra were unavailable. The number and positions of incorporated deuterium atoms revealed the sequence of enzymatic reactions, distinguished double-bond reduction from ketone reduction, and localized reduction sites to the A-ring.

Applying this approach based on pooled fecal inoculum to three steroid substrates enabled reconstruction of their reductive cascades. Progesterone and 19-hydroxy-4-androstene-3,17-dione metabolism proceeds through sequential two-step reduction: first the C4–C5 double bond (two deuterium labels), then C3 carbonyl (one label). For 17α-hydroxypregnenolone, a three-step pathway was elucidated, that is, initial 3β-oxidation/Δ^5−4^ isomerization (one deuterium label), followed by the same two reduction steps as progesterone and 19-Hydroxy-4-androstene-3,17-dione, resulting in up to four deuterium labels confined to the A-ring.

Beyond mechanistic insights, the D_2_O-labeling workflow holds potential for clinical translation. The gut microbiome functions as a virtual endocrine organ, modulating host steroid hormone levels through enzymatic deconjugation, enterohepatic circulation, and reductive transformations. Dysregulation of this microbial steroid metabolism has been implicated in endocrine disorders, inflammatory bowel disease, and neuropsychiatric conditions. The approach described here could be deployed to monitor microbial steroid-converting activity in patient cohorts, assess interindividual variability in drug metabolism, and evaluate the efficacy of steroid-based therapies. While direct clinical correlations remain to be established, this workflow provides a practical tool for bridging mechanistic microbiology with clinical endocrinology and pharmacology, and for guiding personalized intervention strategies targeting the host–microbiota steroid metabolic network.

## Figures and Tables

**Table 1:** Characteristics of compounds detected during incubation of progesterone with gut microbiota.

Compound	R_t_, Min	Ion Type, *m*/*z*	Number of D Atoms
Progesterone	9.17	[M+H]^+^, 315.2319	0
Dihydroprogesterone	9.88	[M+H]^+^, 317.2475–319.2601 [M+H-H_2_O]^+^, 299.2370–301.2495	0–2
Pregnanolone	9.55, 9.79	[M+H]^+^, 319.2632–322.2820 [M+H-H_2_O]^+^, 301.2526–304.2714	0–3

**Table 2:** Characteristics of compounds detected during incubation of 19-hydroxy-4-androstene-3,17-dione with gut microbiota.

Compound	R_t_, Min	Ion Type, *m*/*z*	Number of D Atoms
19-hydroxy-4-androstene-3,17-dione	5.30	[M+H]^+^, 303.1955	0
19-hydroxyandrostan-3,17-dione	5.07	[M+H]^+^, 305.2111–307.2237 [M+H-H_2_O]^+^, 287.2006–289.2131	0–2
3,19-dihydroxyandrostan-17-one	4.83; 5.54	[M+H-H_2_O]^+^, 289.2162–292.2350	0–3

**Table 3:** Characteristics of compounds detected during incubation of 17α-hydroxypregnenolone with gut microbiota.

Compound	R_t_, Min	Ion Type, *m*/*z*	Number of D Atoms
17α-hydroxypregnenolone	7.69	[M+H-2H_2_O]^+^. 297.2214	0
17α-hydroxyprogesterone	8.41	[M+H-H_2_O]^+^. 313.2162–314.2225	0–1
17α-hydroxypregnanedione	8.21	[M+H-2H_2_O]^+^. 297.2213–300.2401	0–3
17α-hydroxypregnanolone	8.13; 8.64	[M+H-2H_2_O]^+^. 299.2369–303.2620	0–4

## Data Availability

Data not contained within this article or the Supplementary Materials are available upon request.

## Supplementary Materials

The following supporting information can be downloaded at https://www.mdpi.com/article/10.3390/ijms27146466/s1.
