# Supplementary material for: D2O-Enabled Chemical Beaconing: Tracking Steroid Metabolism by Pooled Gut Microbiota
Source: Int J Mol Sci. 2026 Jul 21;27(14):6466. doi: 10.3390/ijms27146466 (PMC13410148; doi:10.3390/ijms27146466)
Supplement: Supplementary file 1 [file ijms-27-06466-s001.zip › ijms-4407878-supplementary.pdf]

Supplementary Materials to the manuscript

D_2O-Enabled Chemical Beaconing: Tracking Steroid Metabolism by Pooled Gut Microbiota

Boris Tupertsev [1,2], Anna Vishnevskaya [1], Tatiana Ikonnikova [1,2] and Yury Kostyukevich [1,*]

[1] Center for Bio- and Medical Technologies, Nobel Str., 3, 121205 Moscow, Russia; btoupersev@gmail.com (B.T.); ai.vish@yandex.ru (A.V.); ikonnikova_tanya@mail.ru (T.I.)
[2] Moscow Center for Advanced Studies, Kulakova Str. 20, 123592 Moscow, Russia
* Correspondence: yura542@gmail.com

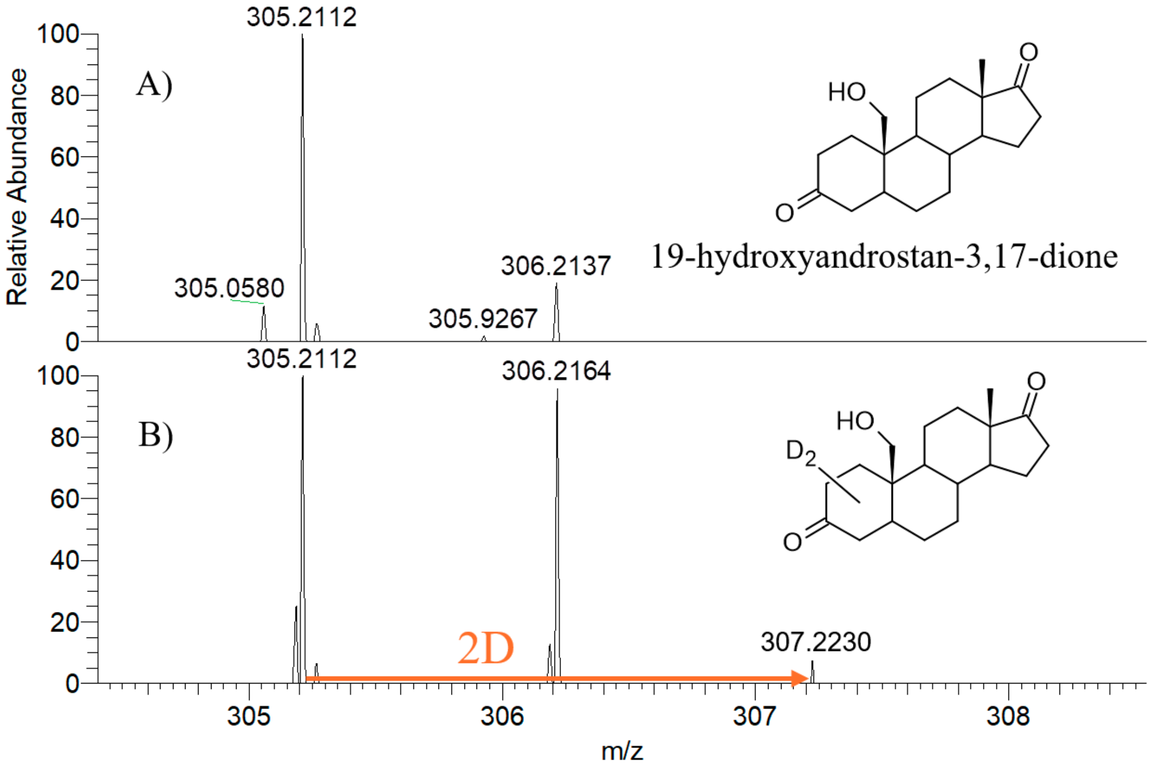

Figure S1. Mass spectra of the compound with retention time 5.07 min: (A) incubation in H_2O; (B) incubation in D_2O

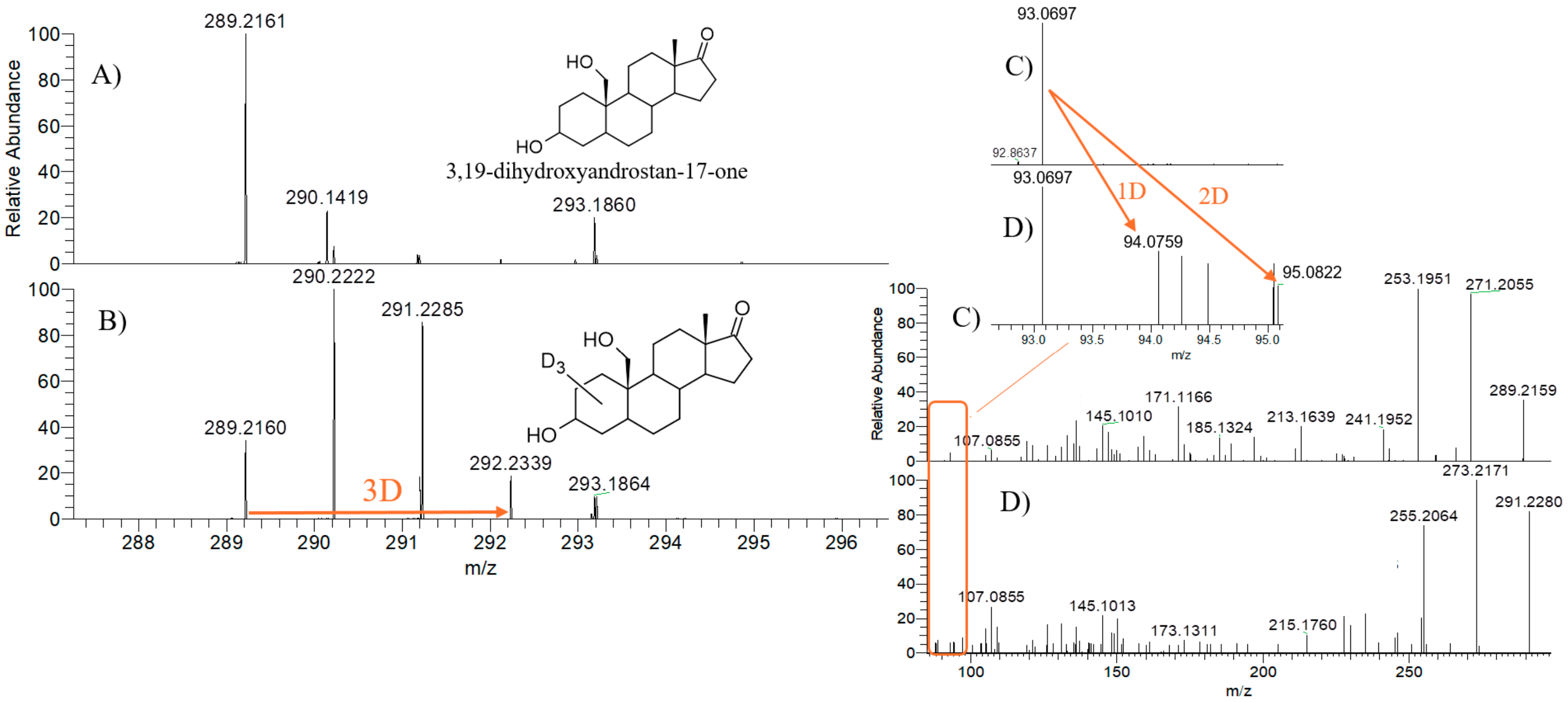

Figure S2. Mass spectra of the compound with retention time 4.83 min (similar for the compound at R_t 5.54 min): (A) MS in H_2O; (B) MS in D_2O; (C) MS/MS of the ion at *m/z* 289.2161 Da in H_2O; (D) MS/MS of the ion at *m/z* 291.2280 Da in D_2O.

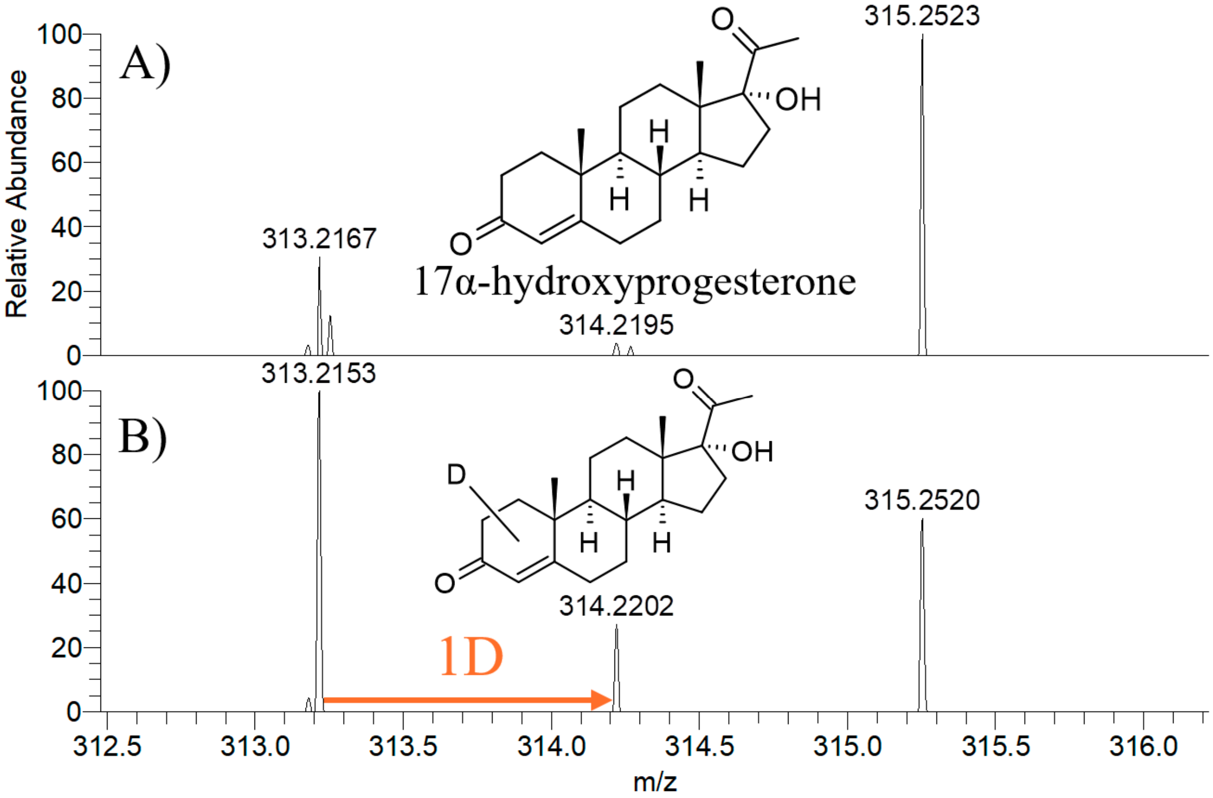

Figure S3. Mass spectra of the compound with retention time 8.41 min: (A) incubation in H_2O; (B) incubation in D_2O

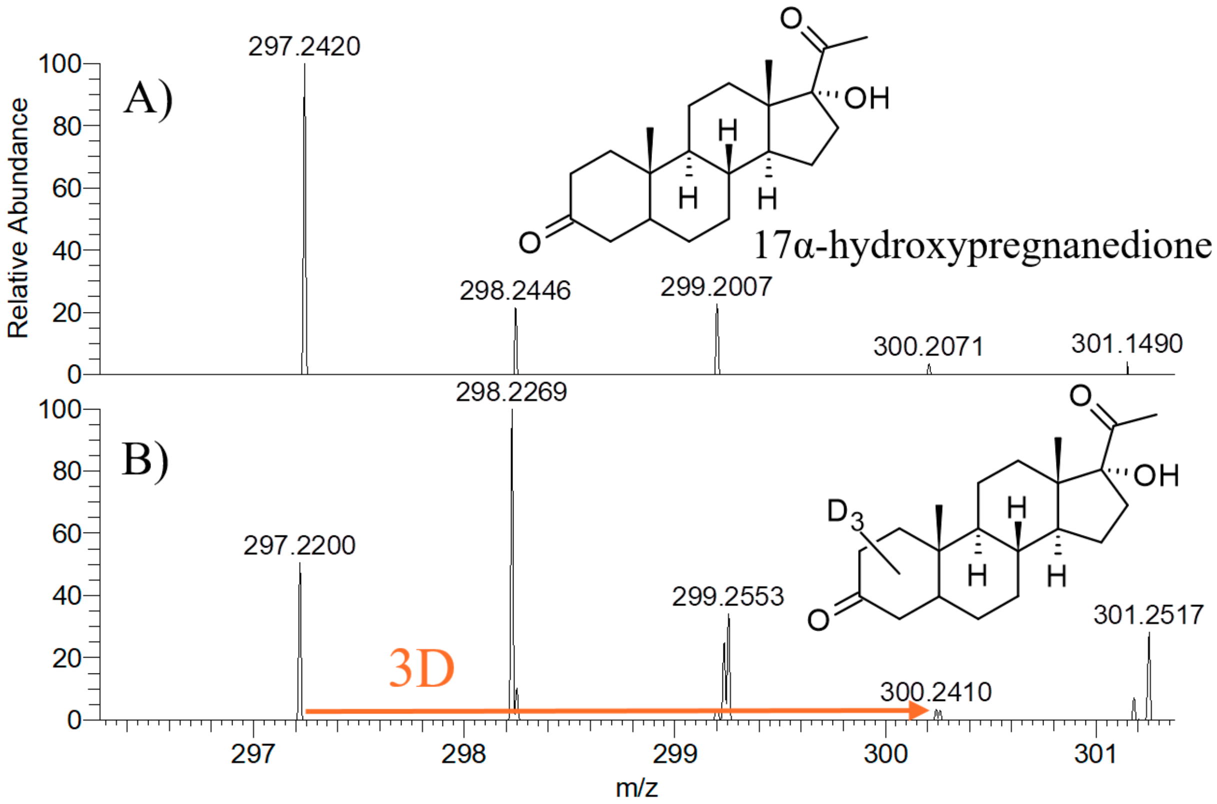

Figure S4. Mass spectra of the compound with retention time 8.21 min: (A) incubation in H_2O; (B) incubation in D_2O
